# Modulatory Potential of Alpinetin on Inflammation, Oxidative Stress, Apoptosis, and Mitochondrial Dynamics in a Rat Middle Cerebral Artery Occlusion Model of Ischemic Stroke

**DOI:** 10.3390/ijms262311329

**Published:** 2025-11-24

**Authors:** Sitthisak Thongrong, Ratchaniporn Kongsui, Lars Klimaschewski, Jinatta Jittiwat

**Affiliations:** 1 Division of Anatomy, School of Medical Sciences, University of Phayao, Phayao 56000, Thailand; sitthisak.th@up.ac.th; 2 Division of Physiology, School of Medical Sciences, University of Phayao, Phayao 56000, Thailand; ratchaniporn.ko@up.ac.th; 3 Department of Anatomy, Histology and Embryology, Division of Neuroanatomy, Medical University of Innsbruck, 6020 Innsbruck, Austria; lars.klimaschewski@i-med.ac.at; 4 Faculty of Medicine, Mahasarakham University, Mahasarakham 44000, Thailand

**Keywords:** ischemic stroke, alpinetin, inflammation, oxidative stress, apoptosis, mitochondrial dynamics

## Abstract

Ischemic stroke initiates a complex cascade of pathophysiological events—including energy failure, excitotoxicity, oxidative stress, inflammation, apoptosis, and mitochondrial dysfunction—that together lead to extensive neuronal damage. Effectively targeting these interconnected mechanisms is crucial for achieving neuroprotection. Alpinetin, known for its antioxidant, anti-inflammatory, and cytoprotective properties, has shown promise as a potential therapeutic agent for cerebral ischemia in preliminary studies. However, the exact molecular mechanisms underlying its neuroprotective effects remain unclear. Therefore, this study aimed to investigate the multifaceted actions of alpinetin in a preclinically relevant right middle cerebral artery occlusion (Rt.MCAO) rat model, focusing on its impact on neuronal survival, inflammation, oxidative stress, apoptosis, and mitochondrial function. Forty male Wistar rats were randomly assigned to four groups: sham operation, Rt.MCAO + vehicle, Rt.MCAO + piracetam (250 mg/kg BW), and Rt.MCAO + alpinetin (100 mg/kg BW). We examined glial cell morphology, protein kinase B (Akt) expression, mitochondrial superoxide dismutase (MnSOD), myeloperoxidase (MPO), anti-apoptotic proteins, mitogen-activated protein kinase (p38 MAPK) and mitofusin-2 (Mfn2). Treatment with alpinetin for 3 days exerted robust neuroprotective effects by significantly reducing astrocytic and microglial activation through the downregulation of glial fibrillary acidic protein (GFAP) and ionized calcium-binding adaptor molecule 1 (Iba-1), restoring Akt expression, decreasing MPO activity, and enhancing MnSOD activity. Additionally, alpinetin modulated apoptotic signaling by lowering pro-apoptotic markers Bcl-2 Associated X-protein (Bax) and caspase-3 while increasing the expression of the anti-apoptotic protein B-cell lymphoma-extra large (Bcl-XL). It also attenuated p38 MAPK activation and preserved mitochondrial integrity by mitigating the decline in Mfn2 levels. Overall, these findings highlight the therapeutic potential of alpinetin in targeting multiple pathological processes involved in ischemic brain injury, supporting its promise as an effective treatment for stroke.

## 1. Introduction

A stroke, frequently called a “brain attack,” happens when the blood flow to a section of the brain is cut off or significantly diminished. This deprives brain tissue of vital oxygen and nutrients, leading to the rapid death of brain cells, often within minutes. This critical medical event is primarily classified into two main categories: ischemic stroke and hemorrhagic stroke. Nowadays, stroke is an incredibly important global health concern because of its major effects on people, healthcare, and national economies. It consistently stands as the second most common cause of death globally and a leading reason for long-term disability, placing a substantial burden on public health [1].

An ischemic stroke occurs when blood flow to a brain region stops abruptly, typically from a thrombus or embolus obstructing a vessel. This deprives brain cells of vital oxygen and glucose, leading to rapid ATP depletion. The resulting energy failure disables crucial ion pumps like Na^+^/K^+^-ATPase, causing brain cells to swell and ultimately rupture due to an influx of sodium, chloride, and water [2]. A significant consequence of this energy deficit is the excessive release of glutamate, triggering excitotoxicity and a massive surge of calcium into neurons [3]. This elevated intracellular calcium activates destructive enzymes, produces reactive oxygen species (ROS) leading to oxidative stress, and impairs mitochondrial function [4,5,6], all contributing to irreversible neuronal damage and cell death via necrosis and apoptosis [7]. The irrevocably damaged area is called the ischemic core. The adjacent area, called the ischemic penumbra, is underperfused but potentially salvageable; this vulnerable zone is further compromised by inflammation involving activated microglia, astrocytes, and infiltrating immune cells [8,9,10]. These activations lead to a significant increase in a protein called ionized calcium-binding adaptor molecule 1 (Iba-1) within microglia [11]. Along with the rise in Iba-1, the microglia undergo a profound change in appearance, shifting from a delicate, branched structure to a rounded, more mobile shape. This morphological change signals their transition to a phagocytic role, where they work to clear away damaged cells and debris. Because Iba-1 levels and microglial shape are so closely tied to this process, they serve as a crucial marker for tracking the severity of neuroinflammation after a stroke [12]. While this initial activation can be beneficial for brain repair, a sustained or excessive response can lead to further neuronal damage. Simultaneously, the apoptotic pathway becomes active, with pro-apoptotic proteins like Bax and caspase-3 promoting cell death [13], contrasted by anti-apoptotic proteins like Bcl-XL, which offer protection [14,15,16,17]. Myeloperoxidase (MPO) acts as an inflammatory marker [18], while mitochondrial superoxide dismutase (MnSOD) functions as an antioxidant against ROS [16,17,19]. Beyond these mechanisms, mitochondrial dynamics, particularly the balance between fusion and fission, are crucial for neuronal survival; mitofusin 2 (Mfn2), a key fusion protein, helps maintain mitochondrial integrity and reduce oxidative stress [20]. Similarly, mitogen-activated protein kinase (MAPK) pathways (ERK, JNK, p38 MAPK) activate during ischemia, influencing inflammation, cell death, and survival [21,22]. Furthermore, the protein kinase B (Akt) pathway plays a critical neuroprotective role by fostering cell survival, inhibiting apoptosis, bolstering anti-apoptotic proteins, and mitigating both inflammation and oxidative stress [23,24]. This complex interaction among inflammatory responses, oxidative stress, programmed cell death, altered mitochondrial dynamics, MAPK signaling, and Akt activation suggests the need for a multifaceted therapeutic approach to achieve neuroprotection and enhance recovery after an ischemic stroke.

Alpinetin is a naturally occurring flavonoid, a class of polyphenolic compounds widely found in various plants, particularly those within the *Zingiberaceae* family. Previous laboratory studies have demonstrated that alpinetin possesses a wide range of pharmacological benefits. These include antioxidant [25,26], antimicrobial [27], anticancer [28,29], and anti-inflammatory [25,30] effects, as well as protective actions on the cardiovascular system [27], lungs [31,32], and liver [27,30]. In a recent study by our group, the neuroprotective potential of alpinetin was evaluated using a rat model of ischemic stroke induced by right middle cerebral artery occlusion (Rt.MCAO). Three doses of alpinetin (25, 50, and 100 mg/kg body weight) were administered to determine its efficacy. The results demonstrated that all treatment doses alleviated cerebral injury, with the most potent protective effect observed at 100 mg/kg. At this dose, alpinetin markedly reduced infarct volume, lowered malondialdehyde (MDA) levels, and enhanced the activities of major antioxidant enzymes, including catalase (CAT), glutathione peroxidase (GSH-Px), and superoxide dismutase (SOD). Moreover, treatment with 100 mg/kg alpinetin significantly attenuated neuroinflammatory responses by downregulating COX-2 and IL-6 expression in both the cortical and hippocampal regions [25]. Therefore, in the present study, we selected an effective dose based on those findings to further investigate the underlying mechanisms. The present study examined how alpinetin 100 mg/kg BW impacts glial cell morphology, Akt expression, MnSOD, MPO, anti-apoptotic proteins, as well as the signaling molecules p38 MAPK and Mfn2.

## 2. Results

### 2.1. Effect of Alpinetin on Ischemic Stroke-Induced Morphological Changes in Astrocytes Within the Cortex and Hippocampus

**Figure 1:**
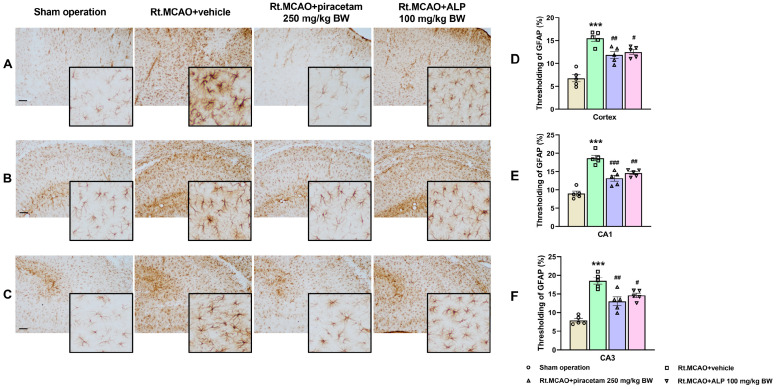
Effect of alpinetin on morphological changes of astrocytes in the cortex and hippocampus. Photomicrographs of brain sections showing GFAP immunohistochemical staining in the infarcted areas of the cortex (**A**), hippocampal CA1 (**B**), and CA3 (**C**). Quantification of GFAP-immunostaining assessed by thresholding analysis in the cortex (**D**), CA1 (**E**), and CA3 (**F**). In the sham operation group, astrocytes exhibit a resting morphology, whereas in the Rt.MCAO + vehicle group, astrocytes display a reactive morphology characterized by extensive branching and hypertrophy, along with an increased GFAP-immunoreactive area indicative of astrocyte activation. Treatment with piracetam or alpinetin reduced astrocyte activation. All images were captured at 10× magnification. Scale bar = 100 µm. The data are represented as mean ± SEM (n = 5). *** *p* < 0.001 vs. sham operation, ^###^ *p* < 0.001, ^##^ *p* < 0.01, and ^#^ *p* < 0.05 vs. Rt.MCAO + vehicle. Rt.MCAO, right middle cerebral artery occlusion; ALP, alpinetin; BW, body weight.

Neuroinflammation commonly triggers the activation of astrocytes. To evaluate this response, immunohistochemistry was used to measure the expression of GFAP, a widely recognized indicator of astrocyte reactivity. As shown in Figure 1, in the sham operation group, astrocytes displayed a normal distribution with minimal GFAP staining observed in both the cortex and hippocampus. Resting astrocytes were characterized by small cell bodies and thin, delicate processes. In contrast, ischemic stroke induced prominent morphological changes in astrocytes, characterized by extensive branching and hypertrophy. This hypertrophy was evidenced by thicker, shorter, and more extensively branched main processes and branchlets. Additionally, ischemic stroke caused a significant increase in GFAP staining intensity (*p* < 0.001). Notably, treatment with alpinetin and piracetam demonstrated a protective effect, as evidenced by the reduced astrocytic activation and GFAP expression observed in the treated subjects compared to those receiving the vehicle.

### 2.2. Effect of Alpinetin on Ischemic Stroke-Induced Morphological Changes in Microglia Within the Cortex and Hippocampus

**Figure 2:**
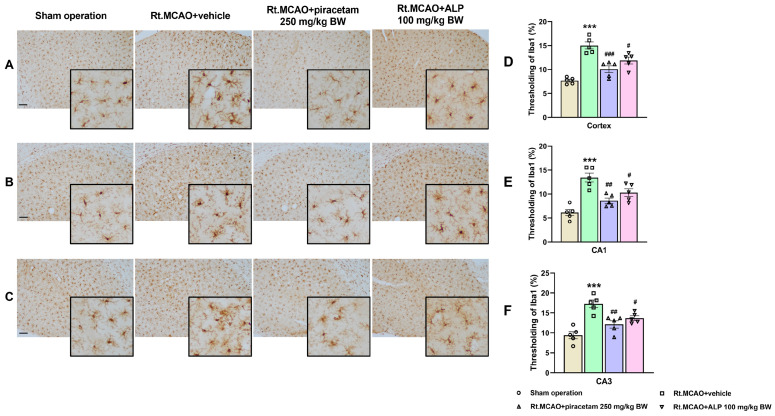
Effect of alpinetin on microglial morphology within the cortex and hippocampus. Iba1 immunostaining on brain section photomicrographs (captured at 10× magnification) was used to visualize microglia across the infarcted cortex, CA1, and CA3 subregions (**A**–**C**). Microglia were observed to shift from a ramified (resting) state in the sham group to an activated, altered morphology with an increased Iba1-positive area in the ischemic (Rt.MCAO + vehicle) group. Quantification confirmed this activation (**D**–**F**). Significantly, treatment with either piracetam or alpinetin diminished microglial activation, suggesting these compounds possess neuroprotective properties. Scale bar = 100 µm. The data are represented as mean ± SEM (n = 5), *** *p* < 0.001 vs. sham operation and ^###^ *p* < 0.001, ^##^ *p* < 0.01, ^#^ *p* <0.05 vs. Rt.MCAO + vehicle. Rt.MCAO, right middle cerebral artery occlusion; ALP, alpinetin; BW, body weight.

Inflammation is a core component in the development of ischemic stroke and is therefore a crucial target for new therapeutic approaches. Among brain-resident immune cells, microglia act as the primary immune cells, rapidly detecting and responding to ischemic injury. They are extremely sensitive to changes in cerebral blood flow, quickly altering their processes and undergoing significant morphological changes. Consequently, assessing alpinetin’s effect on this microglial transformation was essential to evaluating its potential role in modulating neuroinflammation and protecting against injury-induced activation. To determine the extent of microglial activation and the inflammatory response, Iba1 immunostaining was performed, and representative images are presented in Figure 2. In the sham operation group, microglia displayed a resting morphology characterized by small somata and thin, ramified processes, with weak immunoreactivity across the cortex and hippocampal CA1 and CA3 areas. Conversely, microglia in the ischemic group exhibited an activated morphology, marked by enlarged soma with shortened and thickened processes similar to amoeboid morphology, and a notable increase in Iba1 staining intensity (*p* < 0.001). Crucially, treatment with piracetam (*p* < 0.01) or alpinetin (*p* < 0.05) noticeably reduced this microglial activation in both the cortex and the hippocampus compared to the vehicle-treated group. This finding suggests that both compounds possess potential neuroprotective effects by suppressing the inflammatory microglial response.

### 2.3. Effect of Alpinetin on Akt Expression in the Cortex and CA1 of Hippocampus

**Figure 3:**
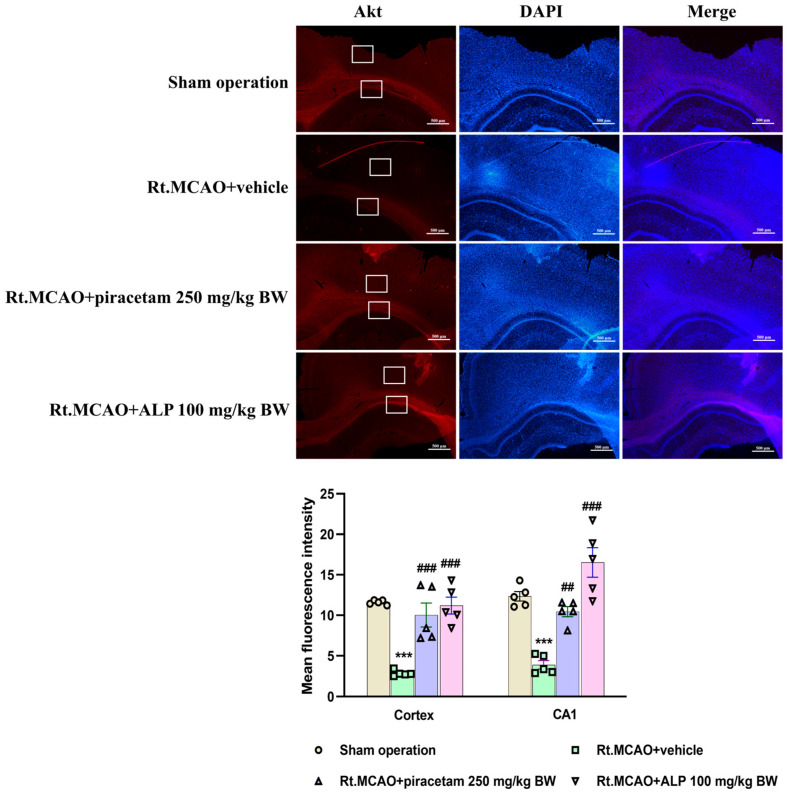
Immunofluorescence labeling of Akt in the cortex and CA1 following MCAO. White boxes indicate the areas analyzed in this study. Quantitative analysis of Akt mean fluorescence intensity revealed a decrease in this intensity in the cortex and CA1 following Rt.MCAO. Notably, alpinetin (100 mg/kg BW) up-regulated Akt expression both in the cortex and CA1. The data are represented as mean ± SEM (n = 5), scale bar = 500 µm. *** *p* < 0.001 vs. sham operation, ^##^ *p* < 0.01, and ^###^ *p* < 0.001 vs. Rt.MCAO + vehicle. Rt.MCAO, right middle cerebral artery occlusion; ALP, alpinetin; BW, body weight.

Akt plays a crucial role in cell survival. Following the MCAO, quantification analysis revealed a significant decrease in Akt expression within both the cortex and hippocampal CA1 (Figure 3). Interestingly, treatment with alpinetin (100 mg/kg BW) reversed the decrease in Akt expression (*p* < 0.001) (Figure 3). These findings suggested that the neuroprotective effect of alpinetin following MCAO is mediated by the up-regulation of Akt expression in the cortex and hippocampal CA1 subfield.

### 2.4. Effect of Alpinetin on Myeloperoxidase (MPO) Activity in Ischemic Brain Tissue

**Figure 4:**
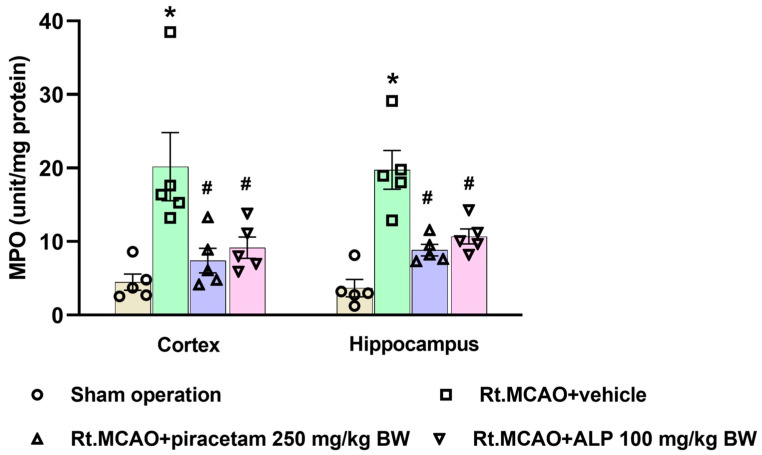
MPO (myeloperoxidase) activity in the cortex and hippocampus of rats subjected to right middle cerebral artery occlusion (Rt.MCAO), with or without alpinetin (ALP) treatment. Data are presented as mean ± SEM (n = 5). Statistical significance was determined by group comparisons: * *p* < 0.05 indicates a significant difference from the sham-operated group, and ^#^ *p* < 0.05 denotes a significant difference relative to the Rt.MCAO + vehicle group. BW = body weight.

Given that Right Middle Cerebral Artery Occlusion (Rt.MCAO) triggers neuroinflammation and subsequent brain injury [33], we assessed key inflammatory pathways by quantifying MPO activity, a common marker for neutrophil infiltration after ischemic stroke. Figure 4 confirms that MPO activity was significantly higher in the Rt.MCAO + vehicle-treated rats compared to the sham-operated controls (*p* < 0.05). Crucially, alpinetin and piracetam administration significantly lowered this heightened MPO activity in both the cortex and hippocampus when compared to the Rt.MCAO + vehicle group (*p* < 0.05).

### 2.5. Effect of Alpinetin on Mitochondrial Superoxide Dismutase (MnSOD) Activity in the Cortex and Hippocampus

**Figure 5:**
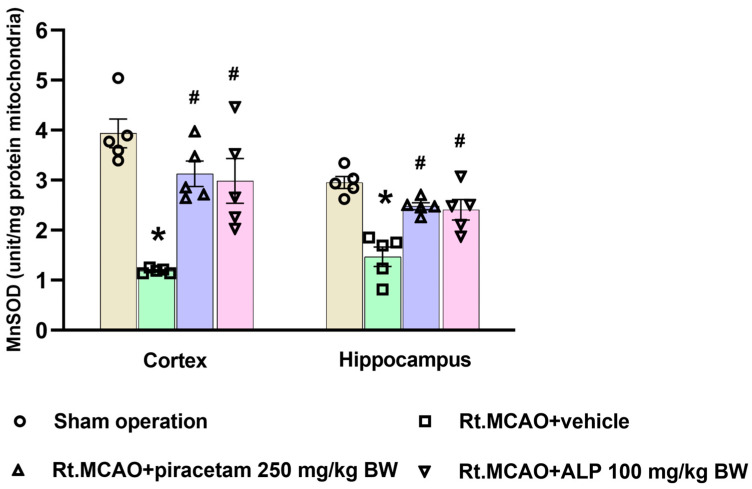
MnSOD activity in rat brain regions following right middle cerebral artery occlusion (Rt.MCAO) and treatment. The graph shows mitochondrial superoxide dismutase (MnSOD) activity in the cortex and hippocampus of rats subjected to Rt.MCAO and treated with vehicle, piracetam, or alpinetin (ALP). Data are presented as mean ± SEM (n = 5). Statistical significance was determined as follows: * *p* < 0.05 versus the sham-operated group; ^#^ *p* < 0.05 versus the Rt.MCAO + vehicle group. BW = body weight.

As shown in Figure 5, Rt.MCAO led to a pronounced reduction in MnSOD activity in the cortex and hippocampus relative to the sham group (*p* < 0.05). Fortunately, this deficit was effectively overcome by the tested compounds. When compared to the Rt.MCAO + vehicle group, animals treated with piracetam (250 mg/kg BW) or alpinetin (100 mg/kg BW) showed significantly higher MnSOD activity (*p* < 0.05).

### 2.6. Effect of Alpinetin on Anti-Apoptotic Signaling in Rt.MCAO-Induced Brain Injury

**Figure 6:**
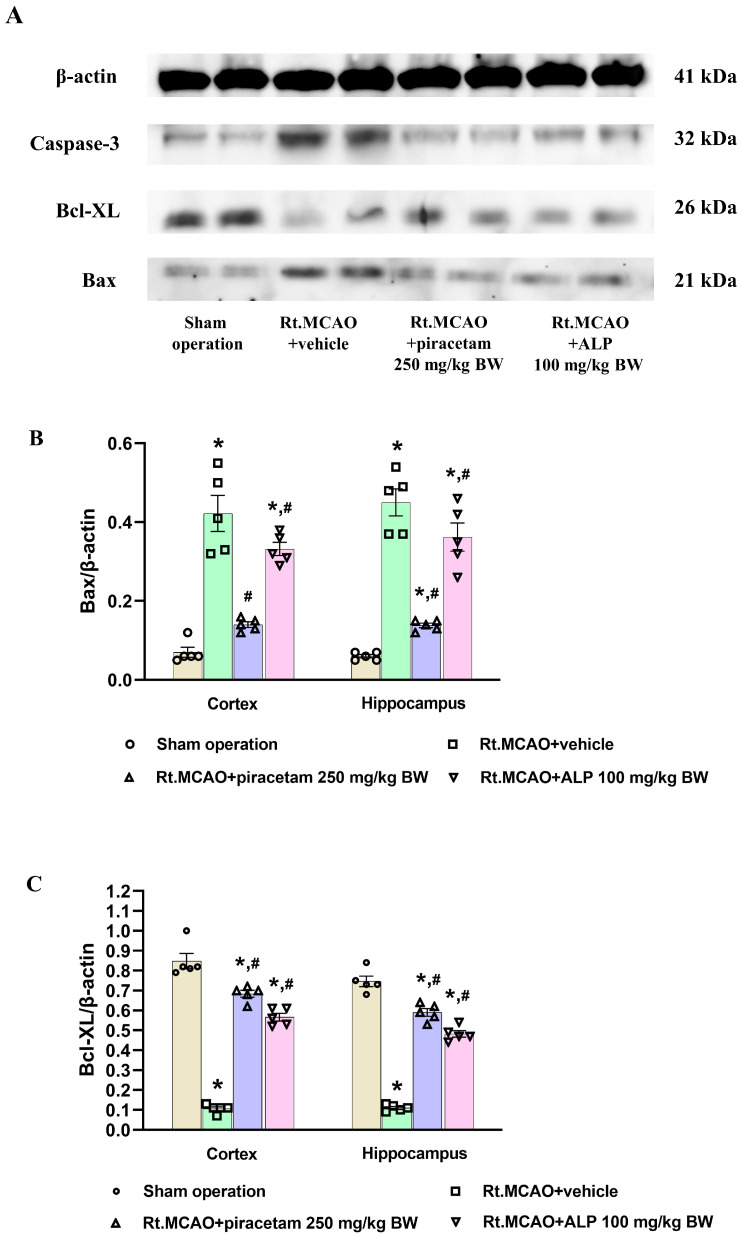

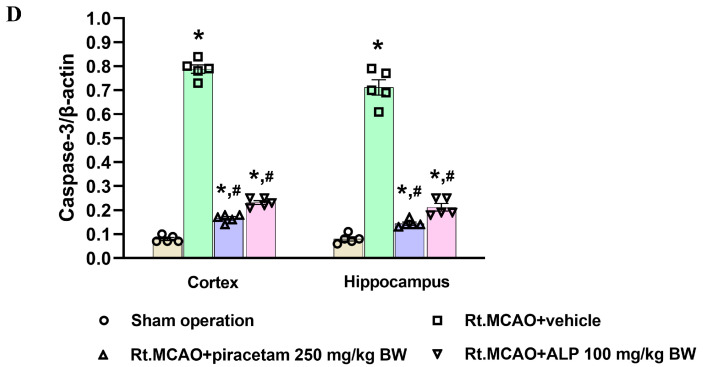
Modulation of apoptotic proteins by alpinetin and piracetam. Western blotting was conducted to determine the protein expression of Bax, Bcl-XL, and caspase-3 in the cortex and hippocampus of rats following right middle cerebral artery occlusion (Rt.MCAO). The comparison includes the sham, Rt.MCAO + vehicle, Rt.MCAO + piracetam (250 mg/kg BW), and Rt.MCAO + alpinetin (ALP, 100 mg/kg BW) groups. (**A**) Immunoblot images from the cerebral cortex confirm the molecular weights of Bax (21 kDa), Bcl-XL (26 kDa), caspase-3 (32 kDa), and the β-actin loading control (41 kDa). (**B**–**D**) Quantitative data for Bax, Bcl-XL, and caspase-3 expression, normalized to β-actin. Each data point is the mean ± S.E.M. (n = 5). Significant differences are as follows: * *p* < 0.05 (relative to the sham group) and ^#^ *p* < 0.05 (relative to the Rt.MCAO + vehicle group). BW = body weight.

The effect of the treatments on apoptosis was investigated by measuring Bax, Bcl-XL, and caspase-3 protein levels in the cortex and hippocampus via Western blotting. Figure 6A shows the distinct bands for β-actin (41 kDa), Bax (21 kDa), Bcl-XL (26 kDa), and caspase-3 (32 kDa). We found a significant drop in the density ratios of the pro-apoptotic proteins, Bax and caspase-3, relative to the β-actin control in the ischemic rats treated with piracetam and alpinetin (Figure 6B,D). Furthermore, the density ratio of the anti-apoptotic protein Bcl-XL to β-actin was significantly higher in these same groups, indicating protection (*p* < 0.05 versus the Rt.MCAO + vehicle group; Figure 6C).

### 2.7. Effect of Alpinetin on p38 MAPK and Mitofusin-2 (Mfn2) Expression Following Ischemic Stroke

**Figure 7:**
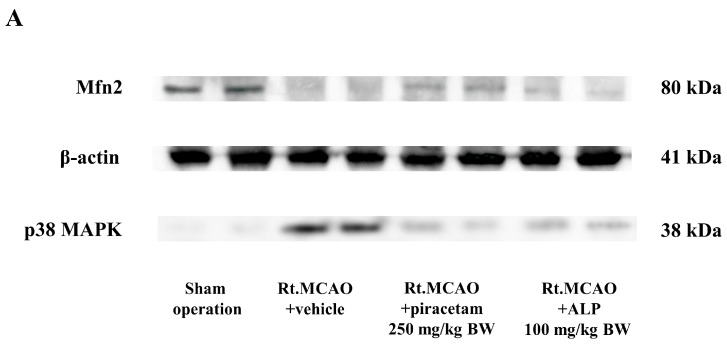

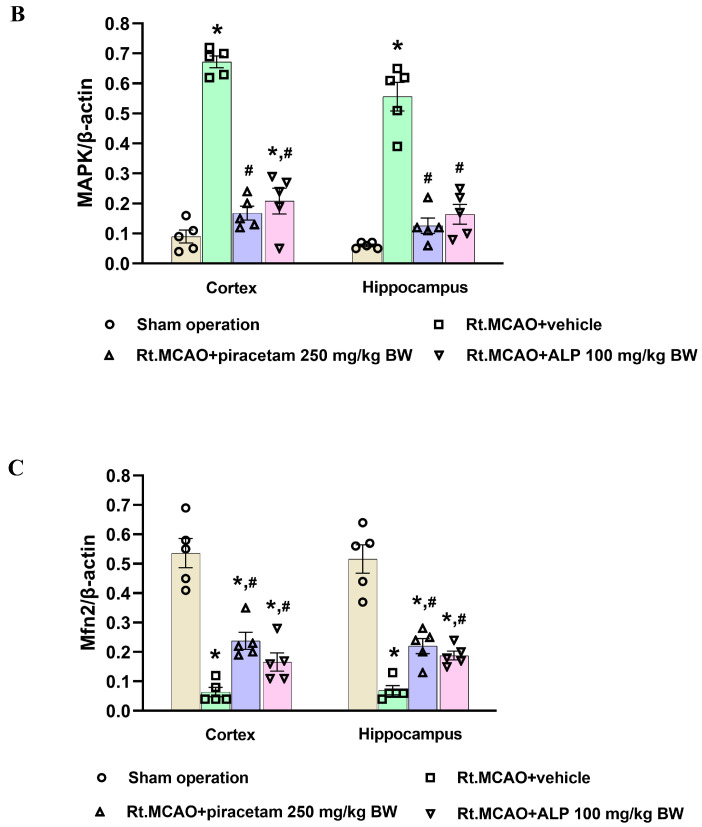
Expression of p38 MAPK and Mfn2 following alpinetin treatment. This figure illustrates the effects of alpinetin on p38 MAPK and Mfn2 expression, determined via Western blot analysis. (**A**) A sample immunoblot from the cortex displays the protein bands for p38 MAPK (38 kDa), Mfn2 (80 kDa), and the β-actin loading control (41 kDa). (**B**,**C**) Quantitative analysis of the band densities for p38 MAPK and Mfn2, respectively, normalized to β-actin, is presented for both the cortex and hippocampus. Data are shown as the mean ± SEM (n = 5). Significance levels are as follows: * *p* < 0.05 compared to the sham operation group; ^#^ *p* < 0.05 compared to the Rt.MCAO + vehicle group. Abbreviations: MAPK, mitogen-activated protein kinase; Mfn2, mitofusin 2; Rt.MCAO, right middle cerebral artery occlusion; BW, body weight; ALP, alpinetin.

To evaluate the role of alpinetin in supporting mitochondrial dynamics against ischemic oxidative stress, we assessed the protein expression of p38 MAPK and Mfn2. The Western blot results (Figure 7A) confirmed the target proteins: p38 MAPK (38 kDa), Mfn2 (80 kDa), and β-actin (41 kDa). Our data reveals that the Rt.MCAO + vehicle group suffered a significant elevation in the p38 MAPK/β-actin ratio and a concurrent reduction in the Mfn2/β-actin ratio compared to the sham group (Figure 7B,C). Piracetam (250 mg/kg BW) and alpinetin (100 mg/kg BW) treatment mitigated these changes effectively. Both treatments resulted in a significant decrease in the p38 MAPK/β-actin ratio (*p* < 0.05; Figure 7B) and significantly preserved the Mfn2/β-actin ratio (*p* < 0.05; Figure 7C) relative to the vehicle-treated animals.

## 3. Discussion

Neuroinflammation is a hallmark of many central nervous system (CNS) pathologies, including ischemic stroke, where the restriction of blood flow leads to rapid neuronal injury and a cascade of cellular and molecular responses. Among the earliest and most prominent responders to ischemic insult are the brain’s resident immune cells—microglia—and the most abundant glial cells—astrocytes. These glial cells are instrumental in orchestrating both the progression of damage and the initiation of repair processes. During the first three days following an ischemic stroke, microglia and astrocytes are key drivers of both injury progression and early healing [12,34]. Microglia become activated quickly, releasing inflammatory factors that intensify neuronal damage and disrupt the blood–brain barrier [12]. Meanwhile, astrocytes undergo reactive astrogliosis, a process identified by heightened production of the structural protein GFAP, which reflects their transition into a reactive state [34]. These astrocytes not only contribute additional inflammatory signals but also engage in cross-talk with microglia, creating a feedback cycle that further aggravates tissue injury. Despite these harmful effects, both cell types simultaneously initiate repair mechanisms: microglia begin clearing cellular debris through phagocytosis, and astrocytes start forming a glial scar that helps protect nearby healthy tissue [12]. As a result, this 72 h acute phase becomes a critical period that determines how severe the brain damage will be and influences the path of recovery. In our study, animals that underwent a sham operation displayed normal astrocytes with minimal GFAP staining in both the cortex and hippocampus. These resting astrocytes were characterized by small cell bodies and thin, delicate processes. In contrast, an ischemic stroke induced significant morphological changes, including increased GFAP immunoreactivity, extensive branching, and hypertrophy. This hypertrophy was evidenced by thicker, shorter, and more complexly branched processes and branchlets. Notably, treatment with alpinetin and piracetam reduced astrocytic activation and GFAP expression compared to the vehicle-treated group, suggesting a protective effect. Furthermore, research has shown that the Iba-1 protein is a crucial indicator of the brain’s response to an ischemic stroke [12]. Its presence increases dramatically as microglia, the brain’s immune cells, change from their usual branched form into an active, rounded state. This change is essential for the cells to migrate to the injured area and get rid of dead tissue [35]. In response to injury, microglia can shift from a resting state to a reactive state, a phenomenon recognized for decades. This transition involves a change in morphology: activated cells develop a larger soma and their processes become thicker, more numerous, and complex than those of ramified microglia. The reactive stage is characterized by the shortening and thickening of these cellular processes, eventually resulting in amoeboid, phagocytic cells [36]. By monitoring Iba-1 levels, scientists can better understand the dual nature of microglia: their capacity to both worsen and help heal brain damage after a stroke [37]. This makes the protein a valuable biomarker for research into the mechanisms of ischemic brain injury and the search for new therapies. Microglia, the immune cells of the brain, are quickly and predictably activated after an ischemic event. Many studies suggest this microglial activation can serve as a measure of the extent or severity of the resulting brain damage [38,39,40]. Additionally, nuclear factor-kappa B (NF-κB) is a pivotal transcription factor that plays a central role in neuroinflammation and subsequent neuronal damage. Activation of NF-κB signaling upregulates the production of pro-inflammatory mediators such as tumor necrosis factor-alpha (TNF-α), interleukin-1 beta (IL-1β), interleukin-6 (IL-6), and inducible nitric oxide synthase (iNOS) [41]. These cytokines amplify the inflammatory cascade, leading to increased oxidative stress, leukocyte recruitment, and neuronal injury [25,41]. A growing body of research indicates that flavonoids can suppress the pro-inflammatory activation of microglia. This effect is achieved by inhibiting the expression of the Iba-1 protein [42,43]. Consistent with prior studies, the current research suggests that alpinetin, a natural flavonoid, can suppress the activation of microglia, thereby helping to reduce brain inflammation after an ischemic stroke. In addition, an inflammatory reaction and oxidative stress are triggered, which significantly worsen brain damage during a stroke. A crucial factor in this process is MPO, an enzyme mainly released by neutrophils, a type of white blood cell [18,44]. While MPO is necessary for the immune system to combat pathogens, its excessive activation after a stroke can be detrimental. The enzyme creates ROS and other harmful substances that can harm the blood–brain barrier and result in widespread oxidative stress and inflammation, which further kills neurons and makes the stroke’s prognosis worse [45]. Consequently, MPO has emerged as a viable therapeutic target for reducing secondary brain damage and a potential biomarker for determining the severity of a stroke [45]. To examine neuroinflammation, we quantified MPO activity—a common indicator of neutrophil infiltration after ischemic stroke. Our results showed that alpinetin and piracetam administration significantly lowered the heightened MPO activity in both the cortex and hippocampus when compared to the Rt.MCAO + vehicle group. Previous studies have also shown that alpinetin attenuates inflammation by reducing MPO activity in a septic mouse model [46] and in mice with lipopolysaccharide (LPS)-induced endometritis [47]. Targeting MPO offers a promising therapeutic strategy for ischemic stroke by addressing several key mechanisms of brain injury. By directly inhibiting this crucial enzyme, therapies can effectively reduce oxidative stress and neuroinflammation, two primary drivers of neuronal death after a stroke [45]. The protective effects of MPO inhibition include preventing the creation of toxic oxidants like hypochlorous acid (HOCl), limiting the infiltration of damaging inflammatory cells, and helping to maintain the integrity of the blood–brain barrier [43]. These combined actions ultimately result in a smaller area of damaged brain tissue, improved neurological function, and a reduced risk of complications [45,48,49]. Therefore, MPO inhibition, particularly when administered during the subacute phase, has significant potential to improve recovery outcomes.

Akt is an essential protein that plays a key role in regulating many cellular processes, especially the crucial balance between cell life and death [50]. It is a central part of a protective signaling network called the PI3K/Akt/mTOR pathway, which is particularly important after a stroke [51]. Following a stroke, the brain is starved of oxygen and glucose, triggering a chain reaction that results in the death of brain cells. In response, Akt becomes active and initiates a defense mechanism [52]. It does this by chemically altering, or phosphorylating, specific proteins that promote cell death. By deactivating these proteins, Akt effectively stops apoptosis, a type of programmed cell death [53]. Moreover, Akt promotes the activation of nuclear factor erythroid 2-related factor 2 (Nrf2), which is the nuclear translocation of the antioxidant transcription factor. Activation of the PI3K/Akt/Nrf2 signaling pathway plays a crucial role in cell survival, helping cells maintain oxidant-antioxidant homeostasis and protecting them from oxidative damage in the ischemic stroke model [50]. This protective action helps brain cells in the affected area survive, which in turn limits the overall damage from the stroke. Understanding the exact mechanisms of Akt’s function could lead to new therapeutic approaches that enhance its neuroprotective abilities. Based on this information, we also determined the effect of alpinetin on the Akt protein using a fluorescence assay. These findings suggest that the neuroprotective effect of alpinetin following MCAO is mediated by the up-regulation of Akt expression in the cortex and hippocampal CA1 subfield. Flavonoids modulate the Akt/PKB signaling pathway, a key regulator of cell survival and growth [54]. Compounds like kaempferol can activate PI3K/Akt, leading to stronger survival signals and reduced apoptosis [54]. This influence on Akt activity contributes to the neuroprotective, cardioprotective, and anticancer effects of flavonoids, suggesting they are promising natural agents for manipulating cell survival mechanisms through this pathway [54]. On the other hand, ischemia activates a process of programmed cell death called apoptosis, leading to ongoing neuronal loss [55]. Ischemia induces an imbalance in apoptotic signaling, where increased expression of Bax and caspase-3, coupled with decreased levels of protective proteins such as Bcl-XL, contributes to neuronal degeneration in susceptible regions like the cortex and hippocampus [16,17,56]. Conversely, anti-apoptotic molecules such as Bcl-2 and Bcl-XL are essential for supporting neuronal viability and reducing infarct size in both transient reperfusion and permanent ischemic stroke models [57,58]. In the present study, we investigated whether alpinetin could exert neuroprotection through its anti-apoptotic mechanism in a rat model of Rt.MCAO. Treatment with alpinetin and piracetam in ischemic rats significantly reduced the pro-apoptotic protein ratios (Bax and caspase-3; Figure 6B,D) and significantly increased the anti-apoptotic protein ratio (Bcl-XL; *p* < 0.05 vs. vehicle; Figure 6C), suggesting effective neuroprotection. A previous study demonstrated that flavonoids, such as puerarin, protect brain tissues after hemorrhagic injuries by modulating apoptotic pathways. They accomplish this by suppressing the pro-apoptotic protein Bax while promoting the anti-apoptotic protein Bcl-2, thereby preventing neuronal cell death [59]. Moreover, the findings by Su et al. [32] suggest that alpinetin may offer a therapeutic benefit for chronic obstructive pulmonary disease (COPD) by addressing both cellular and inflammatory aspects of the disease. Their study indicates that alpinetin inhibits the apoptosis of alveolar cells by suppressing the activity of caspase-3 and caspase-9 [32]. Our previous publication [25] using the same experimental model demonstrated that alpinetin markedly ameliorated neuronal loss in the ischemic cortex and hippocampus, as evidenced by cresyl violet (Nissl) staining. These findings strongly support the neuroprotective potential of alpinetin in ischemic stroke. Furthermore, although we have established alpinetin’s capacity for neuroprotection, the specific molecular targets and underlying signaling cascades require further definition. To gain a comprehensive understanding of how programmed cell death is regulated, subsequent studies should combine TUNEL staining with the analysis of apoptosis-related protein expression. This will be key in accurately identifying neuronal apoptotic events. We recognize the importance of these mechanistic questions and identify them as critical avenues for future research, even though they fall outside the parameters of our present work.

Mitochondria are highly dynamic organelles that sustain cellular energy demands and homeostasis through coordinated processes such as fission, fusion, biogenesis, and mitophagy [60]. The regulation of these mitochondrial dynamics during a stroke is critically managed by various proteins and signaling cascades [21]. Mfn2 is a key mediator of mitochondrial fusion, ensuring structural integrity and stable function of the organelle. A decrease in Mfn2 levels can impair energy production, boost oxidative stress, and disrupt calcium regulation, all of which contribute to worsened neuronal damage [61,62]. Concurrently, signaling cascades like the MAPK pathway modulate the balance between fusion and fission proteins under ischemic stress, altering the organelles’ shape and function [63]. Preserving Mfn2 activity together with appropriate MAPK regulation is therefore essential for maintaining mitochondrial quality control, promoting neuronal survival, and may offer a promising therapeutic avenue for mitigating stroke-related brain damage [15,20,64]. Our research explored how alpinetin influences the expression of p38 MAPK and Mfn2 proteins, given that mitochondrial dynamics can mitigate the damage from cerebral ischemia and oxidative stress. We used Western blot analysis to measure these changes. Both alpinetin and piracetam treatments significantly decreased the p38 MAPK/β-actin ratio (*p* < 0.05; Figure 7B) and preserved the Mfn2/β-actin ratio (*p* < 0.05; Figure 7C) compared to the vehicle-treated animals. This outcome demonstrates their protective effect by maintaining mitochondrial homeostasis against ischemic oxidative stress. A previous study from our group found that galangin, a flavonoid, notably lowered the p38 MAPK to β-actin band density ratio and mitigated the decrease in the Mfn2 to β-actin ratio when compared to the vehicle-treated group [20]. Furthermore, when mitochondrial function is disrupted, it amplifies oxidative injury and, in turn, accelerates neuronal death [63]. MnSOD represents one of the cell’s primary antioxidant defenses, residing within the mitochondria to neutralize oxidative stress [65]. It actively converts the highly reactive superoxide radical into the less harmful hydrogen peroxide. When a stroke causes ischemia, the activity of MnSOD drops, causing this defense to fail. Without it, ROS accumulate uncontrollably, damaging mitochondrial components, halting energy production, and ultimately triggering cell death [66,67,68,69,70]. Therefore, targeting and improving MnSOD activity is a critical therapeutic approach to protect neurons and limit brain damage from a stroke. A marked decrease in MnSOD activity was detected in the cortex and hippocampus following the Rt.MCAO when compared with the sham-operated animals. Treatment with either piracetam (250 mg/kg BW) or alpinetin (100 mg/kg BW) effectively mitigated this reduction, as MnSOD activity levels were significantly elevated in both treatment groups relative to the Rt.MCAO + vehicle group. Flavonoids are a group of natural compounds that can influence several mitochondrial processes, including apoptosis, necrosis, and antioxidant defense [70]. They primarily enhance a cell’s ability to fight oxidative stress by increasing the activity of key antioxidant enzymes. These include heme oxygenase-1 (HO-1), NADPH quinone oxidoreductase-1 (NQO1), superoxide dismutase (SOD2), peroxiredoxin (PRX), glutathione peroxidase (GPX), and catalase (CAT) [71]. Additionally, these compounds can reduce the formation of ROS directly at complexes I and III of the electron transport chain [71].

Piracetam exhibits neuroprotective effects in experimental models of stroke and ischemic brain injury, primarily by reducing oxidative stress and preserving mitochondrial function [20,72,73]. Studies have shown that it mitigates oxidative injury in microglial cells and mouse brains under neuroinflammatory conditions [74] and suppresses neuroinflammation in vascular dementia models [75]. Its benefits also include enhancing mitochondrial dynamics, promoting ATP production, improving cerebral blood flow, and inhibiting apoptosis, thereby limiting neuronal death after ischemic insults [15,20,73,76]. These combined effects make piracetam a suitable positive control for evaluating neuroprotection in our study.

The neuroprotective effects of alpinetin, particularly at a dose of 100 mg/kg, in a rat model of ischemic stroke appear to arise from its multifaceted regulation of key pathophysiological processes. Alpinetin has been shown to exert strong antioxidant effects by enhancing the activity of enzymes such as CAT, GSH-Px, and SOD [25,26,27]. Consistent with these reports, our study demonstrated that alpinetin preserves MnSOD levels, protecting mitochondria from oxidative damage and reducing lipid peroxidation, as reflected by decreased MDA levels [25,77]. In addition to its antioxidant activity, alpinetin exhibits potent anti-inflammatory effects. Treatment with alpinetin attenuated the neuroinflammatory cascade by reducing MPO activity, a marker of neutrophil infiltration, and suppressing glial activation in both microglia and astrocytes, which are key contributors to secondary injury following ischemia. These results are in line with previous studies showing that alpinetin significantly decreases MPO activity, as well as IL-6 and COX-2 expression [25,46]. Alpinetin also modulates apoptotic pathways to promote neuronal survival. In our study, the compound increased the expression of the anti-apoptotic protein Bcl-XL while reducing the pro-apoptotic protein Bax, ultimately suppressing effector caspase activity, including caspase-3. Similar anti-apoptotic effects have been observed in alveolar cells, where alpinetin reduced caspase-3 and caspase-9 activity [32]. Furthermore, Pan et al. [78] reported that alpinetin can inhibit MAPK signaling activation in hepatic ischemia/reperfusion injury, indicating broader cytoprotective properties. Mitochondrial protection is another crucial aspect of alpinetin’s neuroprotective profile. The compound maintained Mfn2 levels, supporting mitochondrial fusion and network integrity, while concurrently reducing p38 MAPK activation, mitigating mitochondrial stress signaling. Collectively, these findings suggest that alpinetin simultaneously targets oxidative stress, neuroinflammation, apoptosis, and mitochondrial dysfunction, providing comprehensive neuroprotection and highlighting its potential as a therapeutic candidate for ischemic stroke. The ability of alpinetin to protect the brain is further supported by its pharmacokinetic and pharmacodynamic properties. Previous research has shown that many flavonoids can penetrate the blood–brain barrier (BBB) primarily through passive diffusion, a process influenced by their lipophilicity—with more lipophilic molecules exhibiting greater permeability. In addition, some flavonoids may access the central nervous system (CNS) via active transport mechanisms mediated by specific influx transporters [79,80,81]. Alpinetin itself has been frequently administered intraperitoneally (i.p.) in experimental models. In various anti-inflammatory and anti-cancer studies, doses ranging from 5 to 100 mg/kg have elicited marked therapeutic responses, including the suppression of inflammatory processes, inhibition of tumor growth, and protection against lung injury [27,77,78]. Evidence from these investigations indicates that i.p. administration provides effective systemic absorption and pharmacological activity, while also bypassing first-pass hepatic metabolism—a notable advantage given alpinetin’s poor oral bioavailability due to rapid glucuronidation [27]. Furthermore, pharmacokinetic data demonstrate that alpinetin administered i.p. at 50 mg/kg exhibits an elimination half-life of approximately 9 h, suggesting prolonged systemic exposure that may facilitate its distribution into the brain [27]. Beyond antioxidant, anti-inflammatory, and anti-apoptotic effects, alpinetin has also been shown to preserve BBB integrity and enhance cerebral blood flow [25,27], further contributing to its overall neuroprotective capacity. Taken together, these findings position alpinetin as a promising natural compound with broad therapeutic potential for ischemic stroke, warranting further preclinical and translational investigation. These multiple benefits suggest that alpinetin has the potential to not only reduce the immediate harm from a stroke but also to aid in long-term recovery, making it a promising candidate for future stroke therapy. Therefore, further studies should investigate whether alpinetin can support long-term recovery after ischemic stroke, beyond its acute neuroprotective effects. Research should focus on its role in processes like neurogenesis, synaptic plasticity, angiogenesis, and glial scar remodeling, as well as its impact on functional outcomes such as motor and cognitive recovery. Long-term preclinical studies with extended treatment and behavioral assessments are needed to determine if alpinetin can promote sustained neurological repair and restoration. Although the current study focused on the most effective dose identified in our previous work, the absence of a dose–response analysis limits the ability to confirm whether the observed neuroprotective effects of alpinetin follow a dose-dependent pattern.

## 4. Materials and Methods

### 4.1. Investigational Compounds

The vehicle used in these experiments was dimethyl sulfoxide (DMSO), obtained from Thermo Fisher Scientific, Inc., Waltham, MA, USA (product code D/4121/PB15). Piracetam, which served as the positive control, was supplied by GlaxoSmithKline Ltd. (Bangkok, Thailand). The compound of interest, alpinetin (a natural dihydroflavone; C_16_H_14_O_4_), was procured from Chengdu Biopurify Phytochemicals Ltd. in Sichuan, China (PubChem ID: 154279). High-performance liquid chromatography (HPLC) analysis verified the purity of alpinetin to be 98%.

### 4.2. Study Design

Eight-week-old male Wistar rats, weighing 250–300 g, were used for the experiments. The rats were sourced from the Northeastern Laboratory Animal Center at Khon Kaen University (Khon Kaen, Thailand). They were housed in groups of five in standard metal cages (37.5 × 48 × 21 cm) and were given a one-week acclimatization period upon arrival. The animals were kept under a controlled 12-h light-dark cycle, with a relative humidity of 30–60% and a stable temperature of 23 ± 2 °C. They had free access to water and commercial pellet food throughout the study. All animal procedures were approved by the Institutional Animal Care and Use Committee at Khon Kaen University, Thailand (approval number: IACUC-KKU-95/64). For this study, forty Wistar rats were divided into four groups as follows:

1. Sham operation group: A sham surgery was performed on these animals, but a nylon filament was not inserted. This group received no subsequent treatment.
2. Rt.MCAO + vehicle group: Following the Rt.MCAO procedure, animals were given an i.p. injection of the vehicle (DMSO) at a volume of 0.5 mL. This treatment was administered daily for three consecutive days to control for the effects of the solvent.
3. Rt.MCAO + piracetam (250 mg/kg BW) group: This group served as the positive control. Animals underwent Rt.MCAO and were subsequently treated with an i.p. dose of piracetam (250 mg/kg BW) once daily for three days.
4. Rt.MCAO + alpinetin (ALP 100 mg/kg BW) group: This group received the Rt.MCAO procedure and was treated with alpinetin at a daily i.p. dose of 100 mg/kg BW for three days.

Upon completion of the 3-day experimental period, five rats from each group were euthanized for immunohistochemical staining of astrocytes, microglia and Akt. For molecular analysis, another five rats per group were used to examine the expression levels of MPO, Bax, Bcl-XL, caspase-3, p38 MAPK, and Mfn2, in both the brain cortex and hippocampus. Additionally, we measured MnSOD activity in the mitochondria of these same brain regions. Based on our previous studies [15,25], we selected the doses of piracetam and alpinetin that demonstrated optimal efficacy, ensuring an appropriate balance between therapeutic effectiveness and safety.

### 4.3. Right Middle Cerebral Artery Occlusion Model

Rats were fasted overnight with free access to water. Anesthesia involved induction with 5% isoflurane and maintenance with 1–3% isoflurane in 100% oxygen. To create permanent focal cerebral ischemia, we employed the intraluminal method to occlude the right middle cerebral artery. A silicone-coated 4-0 monofilament (USS DGTM; Tyco Healthcare Group LP, Norwalk, CT, USA) was inserted into the internal carotid artery and advanced until resistance was felt (approximately 17 mm) [82]. The incision was then closed with sutures and disinfected (10% povidone-iodine). Sham-operated rats received the identical procedure but without filament insertion. Humane endpoints were established (e.g., >20% weight loss, severe symptoms, infection, etc.), but all animals survived the entire 4-day study duration.

### 4.4. Immunohistochemistry Staining for Microglia and Astrocytes

Morphological changes in microglia and astrocytes were assessed using immunohistochemical staining. Free-floating 30 µm PFA fixed sections were processed following a previously described protocol [83]. The tissue sections were first rinsed and treated with 3% hydrogen peroxide (H_2_O_2_) to inhibit endogenous peroxidase activity. To prevent non-specific antibody binding, they were subsequently incubated with 3% normal horse serum (A9647, Sigma-Aldrich; Merck KGaA, Darmstadt, Germany). The sections were then exposed overnight at 4 °C to the primary antibodies: mouse anti-Iba1 (1:250, MABN92, Sigma-Aldrich; Merck KGaA, Darmstadt, Germany) and anti-GFAP (1:300, MAB5628, Sigma-Aldrich; Merck KGaA, Darmstadt, Germany). After washing in 0.1 M phosphate-buffered saline (PBS), the sections were incubated for 2 h at room temperature with a biotinylated donkey anti-mouse secondary antibody (1:500, 715-065-150, Jackson Immuno Research Europe, Ltd., West Grove, PA, USA). This was followed by incubation with extravidin–peroxidase (1:1000, E2886, Sigma-Aldrich; Merck KGaA, Darmstadt, Germany) at room temperature and an additional rinse. Immunoreactivity was visualized using a nickel-enhanced 3,3′-diaminobenzidine (DAB) substrate (D12384, Sigma-Aldrich; Merck KGaA, Darmstadt, Germany). After staining, the sections were rinsed, mounted on positively charged slides, dehydrated through a graded ethanol series, cleared with xylene, and sealed with a coverslip using a mounting medium. Quantitative assessment of GFAP and Iba1 expression was conducted using ImageJ software (version 1.53; National Institutes of Health, Bethesda, MD, USA) with the thresholding analysis function. This method was chosen for its effectiveness in distinguishing specific immunostaining from background signals [84]. The results were expressed as the percentage of the thresholded area.

### 4.5. Immunofluorescence Staining of Akt

Rat brain sections were washed with PBS (three times for 5 min each) and then permeabilized with 0.1% Triton X-100 for 5 min. Subsequently, sections were blocked with blocking solution (10% goat serum in PBS) for 30 min at room temperature. Thereafter, the sections were incubated with Akt1/2/3 primary antibody (1:200, AF6261, Affinity Biosciences, Cincinnati, OH, USA) overnight at room temperature. The sections were then incubated with secondary antibody, Alexa Fluor 594 (1:200, A-11012, Invitrogen, Waltham, MA, USA) for 2 h at room temperature. Subsequently, the nuclei were counterstained with DAPI (1:5000, D9542, Sigma-Aldrich, St. Louis, MO, USA) for 30 s before the sections were mounted with Mowiol 4-88 mounting medium (81,381, Sigma-Aldrich, St. Louis, MO, USA). All brain images were taken by an ECLIPSE Ni-U Upright Microscope (Nikon Corporation, Tokyo, Japan), using the same exposure time for all sections. The Akt intensity measurements in the cortex and CA1 hippocampal subfield were expressed as mean fluorescence intensity after subtraction of the average background, using ImageJ software (Pierce, Rockford, IL, USA).

### 4.6. Protein Quantification

After perfusion, we quickly removed the rat brains and dissected them to obtain the cerebral cortex and hippocampus. Protein levels within these isolated tissues were determined using the Lowry method [85], referencing bovine serum albumin (MilliporeSigma) as the standard. The quantification procedure involved diluting the tissue samples and combining them with a fresh solution of Lowry reagent (containing sodium carbonate, copper sulfate, and sodium potassium tartrate). Following a 10 min incubation at room temperature, diluted Folin–Ciocalteu reagent (1:1 with distilled water) was added. The resulting mixture was incubated for an additional 30 min for color to develop, and absorbance was measured at 650 nm using a spectrophotometer.

### 4.7. Measurement of Myeloperoxidase (MPO) Activity

We quantified MPO activity using a colorimetric assay kit purchased from MilliporeSigma (MAK068). The procedure began by homogenizing brain tissue in four volumes of MPO assay buffer, followed by centrifugation at 13,000× *g* for 10 min at 4 °C to pellet insoluble material. The collected supernatant was loaded onto a 96-well plate in volumes ranging from 1–50 µL, and the final volume in each well was adjusted to 50 µL with MPO assay buffer. Next, a 50 µL master reaction mix was added to all samples, blanks, and controls. The plate was incubated at room temperature in the dark. The reaction was monitored at 30, 60, and 120 min, with 2 µL of stop mix added at each time point. After a 10 min incubation to halt the reaction, 50 µL of TNB reagent was introduced to each well. MPO activity was then determined by measuring absorbance at 412 nm following a final 10-min incubation, comparing sample values to a separately prepared TNB standard curve. Results are expressed in unit/mg of protein.

### 4.8. Brain Mitochondrial Extraction for MnSOD Analysis

After perfusion, the cerebral cortex and hippocampal tissues were rapidly excised for mitochondrial isolation following a previously established protocol from our laboratory [81]. Briefly, the freshly dissected samples were homogenized in mitochondrial isolation buffer and centrifuged at 1000× *g* for 2 min at 4 °C. The supernatant obtained was retained, while the pellet was re-suspended in 0.2 mL of the same buffer and subjected to another centrifugation under identical conditions. Both supernatants were pooled, and 0.07 mL of 80% Percoll (MilliporeSigma) was added. A 0.7 mL layer of 10% Percoll was carefully overlaid on the mixture, which was then centrifuged at 18,500× *g* for 10 min to obtain the mitochondrial fraction. The resulting pellet was washed by re-suspension in 0.7 mL of washing buffer and re-centrifuged at 10,000× *g* for 5 min. The purified mitochondria were finally suspended in washing buffer and stored at −80 °C until further analysis. Manganese superoxide dismutase (MnSOD) activity was subsequently determined using a commercial SOD assay kit (cat. no. 19160; MilliporeSigma, St. Louis, MO, USA), and the results were expressed as units per milligram of mitochondrial protein.

### 4.9. Determination of Mitochondrial Superoxide Dismutase (MnSOD) Activity

MnSOD activity in the cortical and hippocampal regions was determined using a commercial assay kit (cat. no. 19160-1K-F; Sigma-Aldrich, St. Louis, MO, USA). All solutions were freshly prepared following the manufacturer’s guidelines. Sample dilutions and control preparations were performed as required. For the assay, each reaction was set up in a 96-well microplate by adding the designated volumes of substrate solution and reaction buffer, followed by the corresponding test samples, standards, or controls. The enzymatic reaction was initiated with the addition of the detection reagent, and the contents were gently mixed to ensure uniformity. The plate was subsequently incubated at 37 °C for 20 min, after which absorbance was recorded at 450 nm using a microplate reader. Each measurement was conducted in duplicate, and enzyme activity was expressed as units per milligram of mitochondrial protein.

### 4.10. Western Blot Analysis

Upon study completion, we assessed the expression levels of Bax, Bcl-XL, caspase-3, Mfn2, and p38 MAPK in the rat cortex and hippocampus via Western blot analysis. Brain tissue was first homogenized in lysis buffer (Thermo Fisher Scientific, Inc., cat. no. 87792, Waltham, MA, USA), and total protein concentrations were determined [17]. Equal amounts of protein (40 µg) were separated using 10% SDS-polyacrylamide gel electrophoresis and transferred to a Hybond-P (PVDF) membrane (GE Healthcare Limited, Amersham Place, Little Chalfont, Buckinghamshire, UK). Membranes were then incubated overnight at 4 °C with specific primary antibodies: mouse monoclonal anti-Bax (1:500, cat. no. BMS163, Invitrogen by Thermo Fisher Scientific, Inc., Waltham, MA, USA), rabbit monoclonal anti-Bcl-XL (1:1000, cat. no. ab32370, Abcam, Cambridge, UK), rabbit monoclonal anti-caspase-3 (1:2000, ab184787, recognizing pro-caspase-3, Abcam, Cambridge, UK), rabbit monoclonal anti-p38 MAPK (1:500; cat. no. A14401; Abclonal Biotech Co., Ltd., Wuhan, China), rabbit monoclonal anti-mitofusin 2 (1:500; cat. no. A12771; Abclonal Biotech Co., Ltd., Wuhan, China) and anti-β-actin (1:5000; cat. No. AC026; Abclonal Biotech Co., Ltd., Wuhan, China). Following the primary antibody incubation, membranes were incubated for one hour at room temperature with the appropriate secondary antibody at a 1:2000 dilution (cat. no. AS063, Abclonal Biotech Co., Ltd., Wuhan, China). We visualized the immunoreactive proteins using Supersignal West Pico chemiluminescent substrate (Pierce; Thermo Fisher Scientific, Inc., Waltham, MA, USA). Band densities were normalized to β-actin, and protein expression levels were quantified with a ChemiDoc™ MP imaging system and Image Lab software (version 6.0.0 build 25, Bio-Rad Laboratories Inc., Hercules, CA, USA).

### 4.11. Data Analysis

All results are presented as the mean ± standard error of the mean (SEM). We conducted statistical comparisons between groups using a one-way analysis of variance (ANOVA), followed by Tukey’s post hoc test. All analyses were run in SPSS^®^ software (version 25; IBM Corp., Armonk, NY, USA), and a threshold of *p* < 0.05 was established for statistical significance.

## 5. Conclusions

In summary, alpinetin acts on multiple critical mechanisms involved in ischemic brain injury, including inflammation, oxidative damage, programmed cell death, and mitochondrial impairment, offering broad-spectrum neuroprotection and highlighting its promise as a therapeutic option to enhance recovery after stroke. However, further research is necessary to fully understand the specific molecular mechanisms underlying its therapeutic actions.

## Data Availability

The original contributions presented in this study are included in the article. Further inquiries can be directed to the corresponding author.
